# Real-time estimation of wildfire perimeters from curated crowdsourcing

**DOI:** 10.1038/srep24206

**Published:** 2016-04-11

**Authors:** Xu Zhong, Matt Duckham, Derek Chong, Kevin Tolhurst

**Affiliations:** 1 Department of Infrastructure Engineering, University of Melbourne, Parkville, Victoria, 3010, Australia; 2 School of Mathematical and Geospatial Sciences, RMIT University, Victoria 3001, Australia; 3 Department of Forest and Ecosystem Science, University of Melbourne, Burnley, Victoria, 3121, Australia; 4 Department of Forest and Ecosystem Science, University of Melbourne, Creswick, Victoria, 3363, Australia

## Abstract

Real-time information about the spatial extents of evolving natural disasters, such as wildfire or flood perimeters, can assist both emergency responders and the general public during an emergency. However, authoritative information sources can suffer from bottlenecks and delays, while user-generated social media data usually lacks the necessary structure and trustworthiness for reliable automated processing. This paper describes and evaluates an automated technique for real-time tracking of wildfire perimeters based on publicly available “curated” crowdsourced data about telephone calls to the emergency services. Our technique is based on established data mining tools, and can be adjusted using a small number of intuitive parameters. Experiments using data from the devastating Black Saturday wildfires (2009) in Victoria, Australia, demonstrate the potential for the technique to detect and track wildfire perimeters automatically, in real time, and with moderate accuracy. Accuracy can be further increased through combination with other authoritative demographic and environmental information, such as population density and dynamic wind fields. These results are also independently validated against data from the more recent 2014 Mickleham-Dalrymple wildfires.

Natural disasters, such as wildfires, have significant impacts upon human lives, critical infrastructure, and delicate environments worldwide. Timely and accurate information about the areas affected by ongoing natural disasters assists emergency responders and the general public to minimize these impacts. However, in the extreme and unexpected circumstances surrounding an emergency, such real-time information may be in scarce supply. Authoritative information sources may suffer from bottlenecks and high latency. At the other extreme, crowdsourced data from sources such as social media may lack the structure and reliability necessary for automated processing.

For instance, in the case of wildfires, there exist no authoritative information sources that can always and reliably generate up-to-date and accurate information about wildfire perimeters. Although the precise boundary of wildfires is highly dynamic and often vaguely known, emergency management processes already use a variety of sensors with the aim of capturing fire progression as accurately as possible. Information from ground-based sensor networks (e.g.^1^,^2^,^3^), high-resolution satellites (e.g.^4^,^5^,^6^), and airborne infrared scanners (e.g.^7^,^8^,^9^) all play an important role in wildfire emergency planning and response. However, none of these authoritative sources, neither individually nor in combination, can be relied upon for real-time, high frequency, and accurate information about wildfire perimeters during an extreme fire event. Similarly, predictive wildfire behavior models (e.g.^10^,^11^,^12^) are inevitably limited in accuracy by the quality of input data. Whichever data source is used to capture information about fire progression, binary maps of burned/unburned areas are used in many operational wildfire management systems (e.g., Vic emergency^13^ and eMap^14^) to present the real-time progression of wildfires to responders and decision-makers. Although wildfires are by nature more complex than a simple boundary, such maps have the advantage of being an intuitive representation of wildfires for a broad audience, including the general public. Such intuitive information has an important role to play in aiding prompt and correct human decision making during an emergency.

By contrast, an emergency event, such as a natural disaster, often triggers a burst of crowdsourced data more rapidly than authoritative official information^15^. Hence, crowdsourcing has been widely investigated for real-time detection and monitoring of natural disasters including floods^16^,^17^, wildfires^18^,^19^,^20^, and earthquakes^15^,^21^,^22^. However, significant filtering, verification, and quality control are required when using most crowdsourced data sources, such as social media, due to the minimal data structure and preponderance of noisy data not directly relevant to the target event, cf.^23^,^24^,^25^.

Although crowdsourced, calls to the emergency services tend to be less noisy and more reliable than many other sources of crowdsourced data, such as social media, as a result of structuring and filtering of call data by trained human operators. Increasingly, emergency services are collating data about emergency calls into machine-readable formats, and distributing anonymized versions of this data publicly. For example, since 2010 anonymized data about emergency calls and other emergency incidents in Victoria, Australia, has been published in real time using a public RSS feed (see Supplementary information A.1 and Supplementary Fig. S1).

Our overall purpose in this paper is to show that curated crowdsourced data from emergency calls, combined with other authoritative data sources, can be used with established data mining techniques to provide automated, continuous, and real-time estimation of wildfire perimeters. Existing wildfire monitoring methods cannot offer real-time monitoring over large spatial extents at comparably low costs. Using online, publicly-available, real-time, and anonymized data about emergency calls results in an extremely low-cost solution which can be readily implemented. Further, the approach takes advantage of the rapidity with which such calls follow an emergency. The results demonstrate the extent to which data about emergency calls can be used to track wildfire evolution over large spatial and temporal extents, and the considerable potential of curated crowdsourcing in disaster management. The tool affords improved latency and greater resilience compared to existing methods, at the cost of moderate levels of accuracy. As a result, we argue that the approach is a valuable supplement to existing wildfire management systems.

We analyze real-time data concerning the location and subject of emergency calls in order to identify spatial and temporal patterns of calls that can be used to estimate the progression of active wildfires. In Victoria, Australia, emergency calls are answered, categorized, and interpreted into structured spatiotemporal incident data by the Emergency Services Telecommunications Authority (ESTA). This incident data includes the timestamp of the call; the approximate spatial coordinate assigned by the operator to the incident location; and the reported incident type (e.g., grass fire, wildfire, non-structure fire, structure fire, rescue, hazmat incident, and so forth). According to the definitions of the incident categories, records with a type of grass fire, wildfire and non-structure fire were deemed related to wildfire events. Our estimator processes only these records and abandons records of other incident types. Since 2010, this incident data has been made publicly available as an RSS feed by the Victorian Country Fire Authority (CFA).

Like any crowdsourced data, emergency call data can be relied upon to generate a “burst” of real-time information about emergency events. For example, Fig. 1 shows the coincidence of the rapid increase in area burned with a burst of emergency calls during the Black Saturday wildfires of February, 2009. Unlike many other types of crowdsourced data, emergency call data is “curated” in the sense that trained operators answer, categorize, and interpret call details to generate structured spatiotemporal data about each incident.

Despite this curation, call data is still subject to many sources of uncertainty. In the case of wildfires, multiple calls from different locations may refer to the same firefront; conversely calls from nearby locations may refer to different firefronts from amongst multiple active wildfires. Even during a significant wildfire event, many calls may relate to other emergency incidents or to minor or unrelated fires. Emergency calls tend to continue to be received for hours or even days after a firefront has passed through a location. Further, in cases where E-911 positioning information is unavailable (such as in Australia), the operator must elicit an incident location from the caller using natural language. Even in cases where *caller* location is automatically available, the operator must usually still estimate the *incident* location, which in the case of fires may be visible from many miles away.

Using established spatiotemporal clustering and shape-reconstruction techniques, our analysis filters and clusters emergency calls based on topic, spatial location, and timing; and then constructs an evolving “footprint” for the wildfire perimeter based on the call locations and timing. The technique can also integrate relevant authoritative demographic and environmental information, such as population density and dynamic wind fields, in order to improve estimation accuracy. Our analysis can operate in real-time, generating near-instantaneous perimeters based on the latest RSS feed data. (See *Methods* for details of the analysis technique.) As an example, Fig. 2 shows the call locations (estimation input), estimated fire locations (output), and reconstructed fire perimeters (ground truth) at three time steps for the Black Saturday wildfires, 2009.

## Results

Accurate “ground truth” information about the evolution of wildfire perimeters does not usually exist for major wildfires, due to the inherent difficulty of capturing this data using conventional means (as discussed above). However, in the case of the 2009 Black Saturday fires, the firefront progression was painstakingly reconstructed by Gellie *et al.*^26^ over a period of two years after the fire. We use this data to evaluate the wildfire perimeters we estimate from emergency call data. Even though our technique was evaluated using historical data, the algorithm was still run in *online* mode, with the analysis accepting data as if in real time with no forward information about the future development of fires or calls.

There were six separate major wildfires tracked on Black Saturday (cf. Fig. 2), which can be detected in parallel by our technique. We can apply two evaluation metrics commonly-used in information retrieval: precision (positive predictive value) and recall (sensitivity)^27^. Precision can be computed as the number of wildfire perimeter estimates that spatially overlap a true wildfire divided by the total number of estimates. Recall is the number of wildfires detected by our estimator divided by the total number of wildfires.

Given appropriately selected parameterization (discussed below), Fig. 3(a) shows the precision and recall for our estimator for the 2009 Black Saturday wildfires. The figure shows that the estimator can rapidly and accurately identify the different wildfires, achieving at best 67% recall (4/6 wildfires detected) with 50% precision (half estimates are false positives).

Figure 3(b) also shows that the trend of total estimated burned area is similar to that of the true area. However, raw precision and recall ignore the exact shape and precise location of wildfires, tending to inflate the apparent level of accuracy. Instead *area-based* precision and recall (see Supplementary information D)^28^ is preferred in evaluating how closely the wildfire perimeter estimates track the true wildfire progression. Area-based precision is the proportion of the estimated fire area that was in actuality burned. Area-based recall is the proportion of the “ground truth” fire area that is captured by the estimates. Figure 3(c) shows the area-based precision and recall for our estimations. Even with this more discerning, shape-sensitive metric, the results show that the wildfire perimeter estimator can perform moderately well, in less than 12 hours rising to an area-based F1-score (the harmonic mean of the area-based precision and recall) of over 47%.

### Integrating authoritative contextual information

The results in Fig. 3 can potentially be improved by integrating contextual information from authoritative sources. For example, two factors expected to influence strongly the results are population density (in more populous areas, a greater number of emergency calls are to be expected^29^,^30^); and wind speed and direction (in general, although not always, wildfires propagate downwind and rate of spread increases with wind speed). Using a simple weighting process described further in *Methods* our technique can integrate information about these factors, and indeed information about any scalar or vector field.

Figure 4 summarizes the effects of integrating population density and wind speed/direction data upon the overall area-based F1-score. The overall area-based F1-score is used as the summary statistic, computed as the harmonic mean of the average precision and recall over all estimation times. The results indicate that integrating population density improves the overall accuracy of the estimations. Inclusion of wind-speed and direction yields a further increase in estimation accuracy in combination with population density data.

Figure 5 depicts the improved results for the Black Saturday wildfires under both population density and wind field weight. Comparing to the results presented in Fig. 3(b,c), inclusion of population density and wind field information greatly reduces false positive areas.

### Parameterization and cross-validation

The behavior and performance of the basic estimator (without using authoritative information) is tuned through six intuitive parameters, discussed in more detail in *Methods*. Broadly, the parameters relate to: the minimum area of a detected fire (*A*_*t*_); the maximum length of any removable edge in the regular polygon representing the wildfire perimeter (*χ*); the size of the temporal “window” in which calls are processed (*τ*); and the minimum number of calls (*minPts*) in the spatial (*ε*_*s*_) and temporal (*ε*_*t*_) neighborhood required for the underlying ST-DBSCAN clustering algorithm to identify a cluster. *minPts* can be set effectively by a simple heuristic to be the nearest integer ≥2 to ln(*N*)^31^, where *N* is the number of calls in the temporal window. Thus the performance of the estimator is affected by the rest 5 parameters.

The results presented in Figs 3, 4, 5 show the best possible performance, derived from the optimal parameterization (i.e., the combination of parameters that yields the highest overall area-based F1-score). However, a full-factorial design of experiment (see Supplementary information C and Supplementary Table S1) revealed that estimator performance was primarily sensitive to just one of these five parameters: *ε*_*s*_. All other parameters were found to have little effect on performance across a broad range of sensible parameters (see Supplementary Fig. S6). Varying *ε*_*s*_ had the effect of controlling the compromise between area-based precision and recall. Higher values of *ε*_*s*_ tended to overestimate the impacted area (increasing recall); lower values tending to underestimate the impacted area (increasing precision).

The specific parameter settings used to generate the optimal results for the Black Saturday wildfires (see Supplementary Table S2) were cross-validated against the more recent and smaller Mickleham-Dalrymple wildfire, which lasted from 9^th^ February, 2014 to 11^th^ February, 2014. Figure 6 shows that applying the Black Saturday parameter settings to the Mickleham-Dalrymple wildfire achieves high recall (more burned area captured by the estimates), but at the cost of lower precision (more false positive area).

The overall area-based F1-score for the cross-validation is a modest 0.28. Subsequently optimizing only *ε*_*s*_ for the Mickleham-Dalrymple wildfire (i.e., leaving the other four parameters unchanged) enables the overall area-based F1-score of the Mickleham-Dalrymple wildfires to reach 0.46, a remarkably high accuracy.

This independent validation supports the broader argument for the effectiveness of the technique, demonstrating the generality of the approach on different wildfires in different years. The *ε*_*s*_ spatial clustering parameter, in general unknown in advance of a particular wildfire, is a defining factor in the accuracy of estimation. However, the performance of the technique appears largely insensitive to the other parameters, and robust enough to changes in *ε*_*s*_, suggesting that generalized default parameter setting may be reasonable, especially given evaluation against further data sets.

### Latency

One of the key advantages of crowdsourced data, such as emergency calls, is the opportunity to use the burst of data that follows a disaster to generate information more rapidly, i.e., with lower latency, than is possible using authoritative data sources. To evaluate the detection latency of newly burned areas, the study area was discretized into a 1 × 1 km raster grid. The latency for each true positive raster cell (i.e., those cells for which the center is contained in both ground truth and estimation) is measured as time difference between when the cell is estimated to be burned and when that cell is in actuality burned (as indicated by the ground truth). Figure 7 shows the histogram of the detection latency for the Black Saturday and Mickleham-Dalrymple wildfires. For the Black Saturday and Mickleham-Dalrymple wildfires, 76% and 96% of true positive cells are detected no later than 1 hour after the cells are burned, respectively.

Note that many cells are detected with a negative latency, i.e., the estimated fire impact is before the ground truth impact. This result occurs for two reasons. First, some erroneous estimates may by chance subsequently be impacted by fire. However, a more significant reason is the limited temporal granularity of our ground truth data, with updates less frequently and more irregularly than estimates. Thus, our estimates in some cases might reasonably be detecting fire impacts before the ground truth updates. The temporal granularity of the Mickleham-Dalrymple wildfire ground truth is much coarser than that of the Black Saturday wildfires, and as a consequence a larger proportion of cells are detected with a negative latency.

### Spatial distribution of errors

In order to study the correlation between the distribution of errors in estimation and population density distribution, the study area was categorized into three classes of population density. The study area was first discretized into a 1 × 1 km raster grid. Each grid cell was then categorized into three classes of population density using the Jenks natural breaks classification method^32^. The classification was conducted over those cells where population density was less than or equal to 30 people/km^2^, which make up the overwhelming majority of all burned cells (97%). Doing so ensured the classification adequately reflected the differences in that majority of lower population-density regions. The classification breaks provided a high goodness of variance fitness (88%) and were used to categorize all the grid cells. For each estimation time, the area-based precision and recall are calculated in three classes of cells. True positive cells are defined as the cells for which the center is contained in both ground truth and estimation. Per-category area-based precision was computed as the number of true positive cells divided by the number of cells for which the center is contained in estimation. Per category area-based recall was calculated as the number of true positive cells divided by the number of cells for which the center is contained in ground truth.

The errors in estimation were found to have a spatial distribution strongly correlated with population density. Figure 8 shows the area-based precision and recall of our estimates for Black Saturday wildfires, categorized into three classes of population density (*d*) (see Supplementary Fig. S7). The results confirm an expected trend of increasing recall (i.e., fewer false negatives) and decreasing in precision (i.e., more false positives) with greater population density^30^.

The per-category area-based precision and recall are not normally distributed. Hence the Kruskal-Wallis test, a two-tailed nonparametric statistical hypothesis test, is applied to test if the effect of population density on the area-based precision and recall is significant. For the area-based precision, each category contains 71 samples. For the area-based recall, the categories *d* ≤ 3.6 and *d* > 13.2 contain 70 samples, and there are 69 samples in the category 3.6 < *d* ≤ 13.2. The results confirm that population density has a significant impact on the area-based precision (*p* = 2.7 × 10^−6^) and recall (*p* = 5.8 × 10^−12^) at the 5% significance level. Follow-up tests illustrate that for the area-based precision, the category *d* > 13.2 is significantly different from the categories *d* ≤ 3.6 (*p* = 1.2 × 10^−6^) and 3.6 < *d* ≤ 13.2 (*p* = 4.7 × 10^−2^) at the 5% significance level. And there is a significant difference between the categories *d* ≤ 3.6 and 3.6 < *d* ≤ 13.2 (*p* = 2.0 × 10^−2^) as well. For the area-based recall, the category *d* ≤ 3.6 is significantly different from the categories 3.6 < *d* ≤ 13.2 (*p* = 4.7 × 10^−9^) and *d* > 13.2 (*p* = 1.5 × 10^−9^) at the 5% significance level. But the difference between the categories 3.6 < *d* ≤ 13.2 and *d* > 13.2 is not significant (*p* = 0.96).

## Discussion

The results of this analysis show that anonymized data about the location, time, and type of emergency call is sufficient to estimate evolving wildfire perimeters with high accuracy and low latency. Further, the technique is automated, real-time, and based on established and well-understood spatiotemporal clustering and shape-reconstruction algorithms. The results also indicate that estimation performance can be improved through integration of static and dynamic authoritative data when available, such as population density and wind speed and direction. The approach might easily be used to integrate other relevant data sources, including data about fire risk factors such as fuel conditions, topography, temperature, and humidity; short-term or real-time population movements; and mobile phone coverage.

While the technique does require parameterization, the results indicate that performance is dominated by the adjustment of only one parameter. Cross-validation of these parameter settings on fires of different sizes and from different years suggests that the approach is reasonably robust to parameter settings, and acceptable results may be obtainable with default parameterization for a wide range of wildfires. In practical applications, it would also be possible to run multiple estimations in parallel, for example with parameterization optimized for major and more common wildfires.

**Figure 1:**
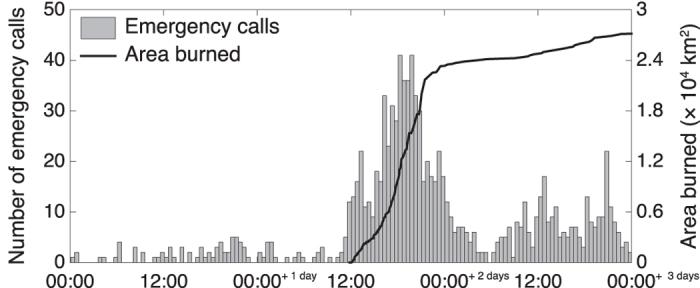
Number of emergency calls (in 30 minutes) and area burned during the Black Saturday wildfires, 7 February 2009.

**Figure 2:**
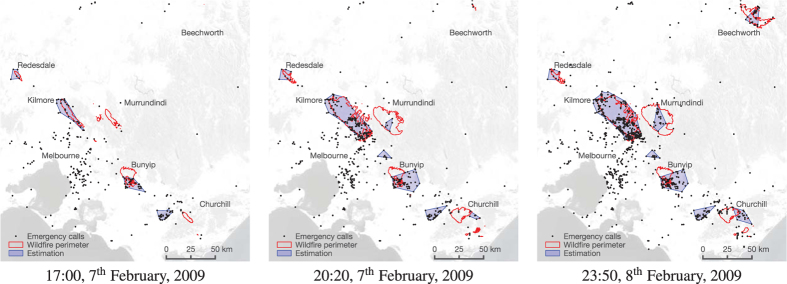
The changing perimeter of the wildfires, together with emergency call locations and perimeter estimations, at three key stages of the Black Saturday wildfires, February 2009. The maps were generated using QGIS 2.8.2-Wien (http://www.qgis.org/en/site/)^52^ and Inkscape 0.91 (https://inkscape.org/en/)^53^.

**Figure 3:**
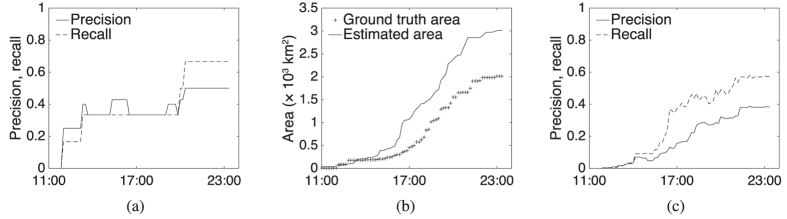
(**a**) Precision and recall of estimates; (**b**) comparison of estimated and true area impacted, and (**c**) area-based precision and recall of estimates for Black Saturday wildfires.

**Figure 4:**
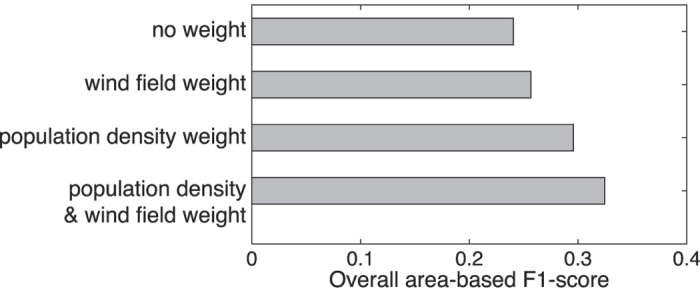
The impact of authoritative information (population density and wind field) on the maximum overall area-based F1-score for estimation of Black Saturday wildfires.

**Figure 5:**
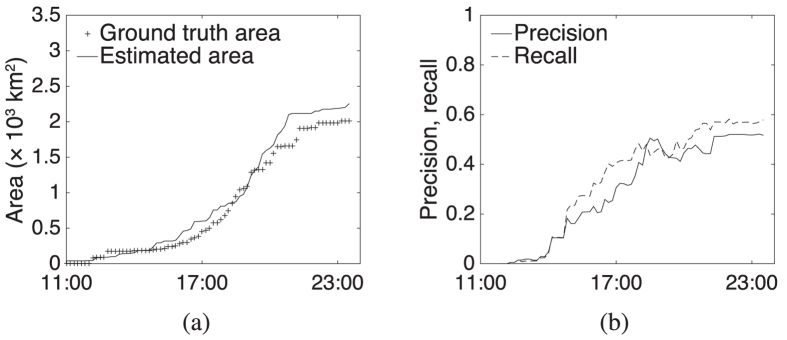
Results for Black Saturday wildfires under both population density and wind field weight: (**a**) comparison of estimated and true area impacted (cf. Fig. 3(b)); and (**b**) area-based precision and recall for estimator (cf. Fig. 3(c)).

**Figure 6:**
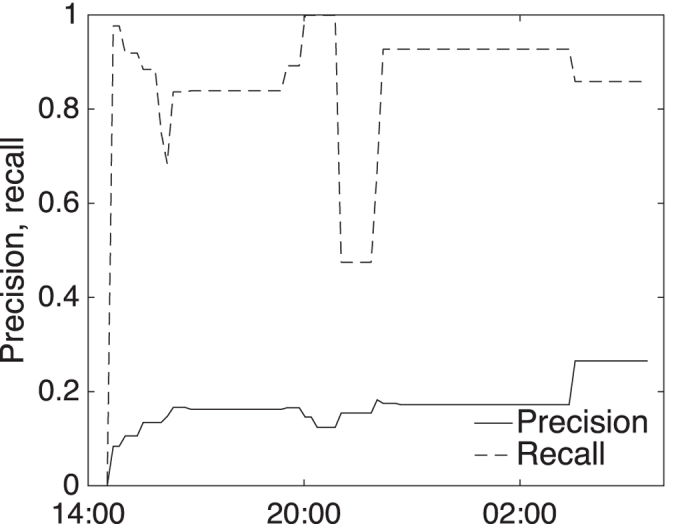
Area-based precision and recall for estimator for Mickleham-Dalrymple wildfires, February 2014.

**Figure 7:**
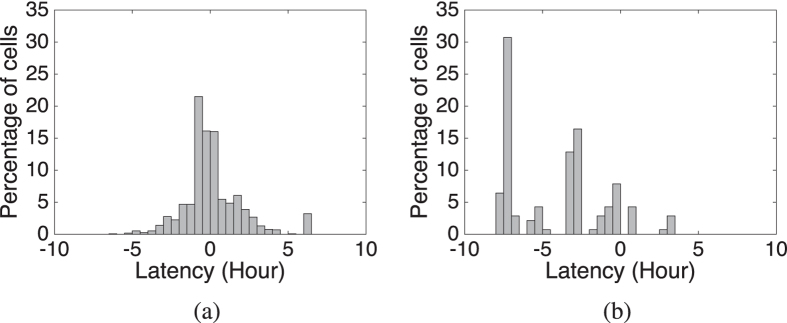
Histogram of the detection latency of (**a**) 1154 true positive cells for Black Saturday wildfires (2009) and (**b**) 140 true positive cells for Mickleham-Dalrymple wildfires (2014). Bar-width is 0.5 hour.

**Figure 8:**
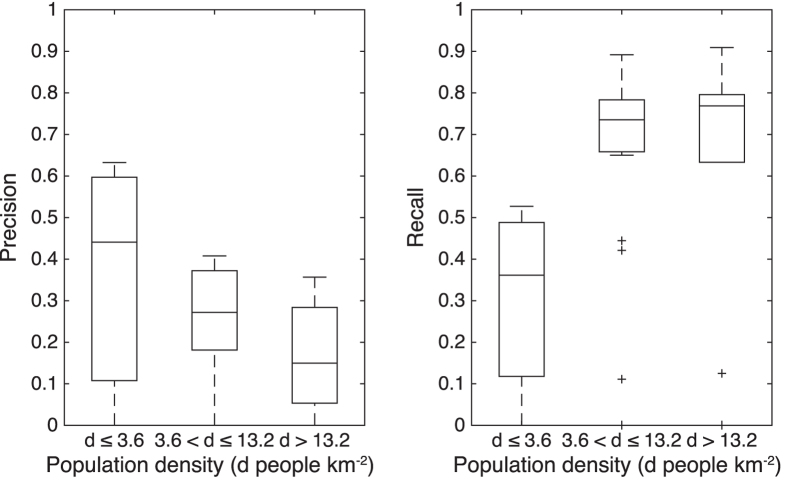
Area-based precision and recall within regions of different population density ranges.

A key advantage of the technique is the ability to take advantage of the “burst” of crowdsourced data that typically follows a disaster, affording reduced latency when compared to existing authoritative data sources. The magnitude of the burst may itself be affected by the emergency. For example, extreme wildfires may lead to telecommunication bottlenecks and failures (as was the case on Black Saturday, where 70% of triple zero calls went unanswered^33^). Nevertheless, emergency events such as extreme wildfires still trigger an abnormally large burst number of emergency calls (cf. Fig. 1), large enough for our estimator to generate valuable results. Hence our estimator is expected to afford greater resilience to bottlenecks and single points of failure than the existing wildfire monitoring methods, even allowing for telecommunications overload and failures.

As expected, errors in estimation were found to be significantly correlated with population density: higher population density generally leads to greater false positives and fewer false negatives. Arguably, false positives are less costly and more acceptable than false negatives for major disasters in populous locations^29^. Thus, within the context of the overall moderate accuracy of the approach, the spatial pattern of errors is compatible with a risk-averse approach to disasters. Errors of omission are more likely in unpopulated locations, where the impact of those errors is likely to be minimized. Errors of commission are more likely in populated locations, where risk aversion makes such errors arguably most acceptable. Hence, the limitations of our approach tend to adapt naturally to the requirements and risk profile of emergency response. In an operational wildfire management system, our estimator might need to be combined with existing authoritative methods to improve the overall situational awareness of wildfires. In particular, the reliance on human crowdsourced contributions means that even allowing for population density variations, our approach is unlikely to ever perform well in the least populous areas. Thus, when a report in the most sparsely populated areas is excluded by our estimator, it may still be necessary for an agency to allocate other resources if available to verification of that report.

Emergency calls reflect the presence of wildfires, but not wildfire absence. Similar presence-only data is familiar to ecologists, for example, in species distribution modeling (SDM)^34^. The performance of naïve analyses of presence-only data, such as simple envelope methods (e.g.^35^,^36^,^37^), is sensitive to biases and outliers in the presence records. More sophisticated techniques, such as generating random pseudo-absence data from background areas (e.g.^38^,^39^,^40^), have been shown to outperform envelope methods^41^. However, the performance of these methods remains sensitive to variations in the quality of pseudo-absence data^42^. Further, as a consequence of the bias of presence-only data, these methods face challenges in the selection of appropriate thresholds for the generation of presence/absence maps (frequently required by practical applications or evaluation, akin to our burned/unburned areas)^43^,^44^. In the case of our estimator, the highly dynamic nature of wildfires makes sampling pseudo-absence data for an active fire event impractical. Consequently, our approach is closer to the naïve envelope methods. However, the spatiotemporal clustering step in our technique helps eliminate outliers from the data. Further, weighting clusters by population density helps reduce the detectability bias in our presence-only data (cf.^34^,^45^). This two-step clustering and non-convex envelope construction affords considerable flexibility^46^, a feature common to superior SDM methods^41^.

Finally, our estimator combines established and well-understood spatiotemporal analysis tools, with proven effectiveness in diverse real-life applications. While our estimates has been tested on data about wildfires, the approach developed in this paper is expected to be easily adaptable to emergency calls concerning a wide range of other disasters characterized by rapidly evolving spatiotemporal extents, such as flooding. Flooding is included in the RSS feeds published by Victoria State Emergency Service (SES)^47^ and South Australian Country Fire Service (CFS)^48^. In practice, though, it may be that flooding generates fewer incident reports than wildfires, and so potentially lowers the efficacy of our crowdsourcing approach. However, our algorithm only requires the input data to be spatiotemporal point observations of an event. So, the approach might also be applied to other types of crowdsourced spatiotemporal data, such as georeferenced social media data, to track the progression of other spatiotemporal events, such as floods. Social media data might improve the performance of our estimator when insufficient emergency calls are received, for example during a flooding event. However, it is challenging to filter and verify social media data, one of the original reasons for examining the important of “curated” crowdsourced emergency calls.

## Methods

### Data

Anonymized data about the type, time, and estimated coordinate location of emergency incidents, including emergency calls, has been published as a public RSS feed by the Victorian Country Fire Authority (CFA) since 2010. This data is near-real time, updated every 5 minutes. Although not publicly available at the time, the emergency call data used in our 2009 Black Saturday evaluation was provided by CFA’s Incident Management System and comprises exactly the same key attributes as those now available in the public RSS feeds. Authoritative contextual data about population density was provided by the Australian Bureau of Statistics (see Supplementary information A.2). The data represents the usual resident population in 1 km^2^ grid format from the 2011 Census of Population and Housing across Australia (see Supplementary Fig. S2). Wind speed and direction were derived from automated weather station (see Supplementary Fig. S4) measurements provided by the Bureau of Meteorology of Australia, interpolated spatially using a widely-used wind model, WindNinja^49^,^50^ (see Supplementary information A.3).

Wildfire perimeter estimates were evaluated through comparison with data about the progression the 2009 Black Saturday wildfires, reconstructed by Gellie *et al.*^26^ drawing on data from a wide range of sources. Data about the perimeters of the Mickleham-Dalrymple wildfires were generated during the course of that 2014 fire using a combination of airborne infrared scanner data and human observations, combined into wildfire perimeters by qualified fire mapping officers. Although these data sets are both subject to significant uncertainty, they are the amongst the best quality data available about any wildfires in Victoria, and so are taken to be our “ground truth.”

### Algorithm

The estimation algorithm itself has four key stages (see Supplementary Fig. S8): incident filtering, spatiotemporal clustering, wildfire perimeter shape reconstruction, and area-based filtering of estimates.

#### Spatiotemporal clustering

The spatiotemporal observations of wildfire positions are clustered using the ST-DBSCAN (spatiotemporal density-based spatial clustering of applications with noise) algorithm^31^. In order to cluster the continuous stream of RSS feed data, ST-DBSCAN was set up to process records within a sliding temporal window of extent *τ* hours, advancing in 10 minutes intervals^51^.

Let *D* be the wildfire reports in the sliding temporal window. Let 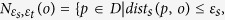 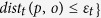 denote the (*ε*_*s*_, *ε*_*t*_)-neighborhood of an object *o* ∈ *D*, where 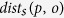 and 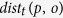 are the Euclidean distance and absolute difference in time between *p* and *o*, respectively. In the standard ST-DBSCAN algorithm, the detection of cluster seeds and the expansion of clusters depend on if 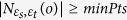, where |(⋅)| is the cardinality of (⋅). In order to handle the expected uneven density of emergency reports, each object *p* ∈ *D* is associated with an empirical weight *w*_*p*_(*p*) which reduces with increasing local population density *d*(*p*). To ensure a symmetric neighborhood relationship between any two objects, the (*ε*_*s*_, *ε*_*t*_)-neighborhood of *o* is modified as 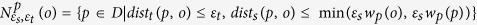. The empirical weight *w*_*p*_(*p*) is calculated as 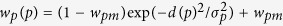, where *σ*_*p*_ > 0 and *w*_*pm*_ ≥ 0 are empirical coefficients. This extension necessitates greater spatial density of calls in more populous locations in order for ST-DBSCAN to identify a cluster (and conversely less dense calls in less populous areas to identify a cluster).

Wind also plays a significant role in wildfire spread. Under both population density and wind field weighting, the modified (*ε*_*s*_, *ε*_*t*_)-neighborhood is defined as 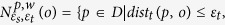 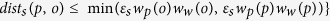, where *w*_*w*_(*p*) is the empirical wind field weight at *p*. The aim of weighting based on wind speed and direction is to encourage the estimates to spread in a downwind direction and restrict the ability of estimates to spread in an upwind direction. Hence weights associated with call locations are increased in the downwind direction of an existing estimation of a wildfire perimeter, and decreased in the upwind direction. Supplementary Fig. S5 illustrates how downwind and upwind objects are determined. In summary, wind-biased outward buffers (see Supplementary information B) are calculated for previously estimated wildfire perimeters using the Huygens’ principle^10^. The wildfire reports inside this buffer are assigned an increased weight 1 + *w*_*rew*_, where *w*_*rew*_ ≥ 0, allowing ST-DBSCAN to identify a cluster based on sparser calls. Conversely, a penalizing unbiased outward buffer of a predefined width *b*_*w*_ (see Supplementary Fig. S5) is calculated for previously detected fire regions with area greater than some threshold *A*_*w*_. Wildfire reports inside the penalizing buffer and outside the wind-biased buffer are deemed to be close to, but in the upwind direction of the previously estimated wildfire perimeters. Hence, calls inside these zones are given a lower weight 1 − *w*_*pen*_, where 0 ≤ *w*_*pen*_ ≤ 1, requiring ST-DBSCAN to encounter greater call density before identifying a cluster. The calls outside the wind-biased outward buffers and the penalizing unbiased outward buffers are not weighted by wind field, i.e., having a wind field weight of 1.

#### Reconstructing wildfire perimeters

The next stage is to reconstruct the shape of individual clusters. The *χ*-shape^46^ algorithm is used for this purpose. However, any shape reconstruction algorithm might easily be substituted for the *χ*-shape. Let 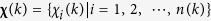 denote the *χ*-shapes of *n*(*k*) clusters in the sliding window at the *k*th epoch. Then candidate wildfire perimeters at the *k*th epoch (**E**_*c*_(*k*)) are calculated as the union of **χ**(*k*) and the candidate wildfire perimeters at the previous epoch (**E**_*c*_(*k* − 1)). At the commencement of the algorithm (*k* = 0), **E**_*c*_(0) = ∅. At the *k*th epoch, **E**_*c*_(*k*) is updated by 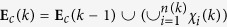. **E**_*c*_(*k*) may occasionally contain holes. Such holes might relate to unburned areas, or to unobserved interior regions of burned areas. We elect to treat holes as undetected burned areas, rather than unburned areas within a fire region because of the inherent bias of emergency call data towards presence. Further, as false positives are less costly and more acceptable than false negatives for major disasters, in the absence of better information it seems reasonable err on the side of caution, and accept a potential bias in our algorithm in the case of holes. Irrespective, this potential bias will be captured by our area-based evaluation metric, which will penalize firefront estimates if they contain unobserved holes.

Finally, the candidate wildfire perimeters **E**_*c*_(*k*) are filtered by area. Only the candidate estimates that exceed a minimum area threshold (*A*_*t*_) are remained as final outputs of the estimator (**E**(*k*)).

## Additional Information

**How to cite this article**: Zhong, X. *et al.* Real-time estimation of wildfire perimeters from curated crowdsourcing. *Sci. Rep.* **6**, 24206; doi: 10.1038/srep24206 (2016).

## Supplementary Material

Supplementary Information

Supplementary Video S1

Supplementary Dataset 1

Supplementary Dataset 2

Supplementary Dataset 3

Supplementary Dataset 4

Supplementary Dataset 5

Supplementary Dataset 6
