# Supplementary material for: Real-time estimation of wildfire perimeters from curated crowdsourcing
Source: Sci Rep. 2016 Apr 11;6:24206. doi: 10.1038/srep24206 (PMC4827086; doi:10.1038/srep24206)
Supplement: Supplementary Information [file srep24206-s1.pdf]

Supplementary information for: Real-time estimation of wildfire perimeters from curated crowdsourcing

Xu Zhong, Matt Duckham, Derek Chong, Kevin Tolhurst

Contents

A Data details **1**
A.1 CFA RSS feeds for emergency calls . . . 1
A.2 Population density data . . . 1
A.3 Wind speed and direction data . . . 1
B Calculating wind-biased outward buffers **1**
C Design of experiment details **2**
D Evaluation details **2**
E Parameterization details **3**
F Supplementary figures and tables **4**
G Legends of supplementary videos and datasets **7**
References **7**

A Data details

A.1 CFA RSS feeds for emergency calls

Emergency calls are answered, categorized, and interpreted into structured spatiotemporal incident data in Victoria, Australia, by the Emergency Services Telecommunications Authority (ESTA). Since 2010, this incident data has been made publicly available as an RSS feed by the Victorian Country Fire Authority (CFA). Before 2010, we made use of archives of the same underlying data generated and stored as part of the Victorian Emergency IMS (Incident Management System).

Fig. S1 shows an example of a CFA RSS feed for wildfire published at 01:49 PM, 10/September/2015. The four attributes used to filter the feed and drive the estimation are highlighted in Fig. S1: "Type," "Start Date/Time," "georss:point," and "guid." According to the definitions of the incident categories, records with a type of "GRASS," "BUSHFIRE," or "NON STRUCTURE" were deemed related to wildfire events and processed by our estimator. The attributes "georss:point" and "Start Date/Time" provide the spatiotemporal coordinates of the observations of wildfire positions. The attribute "guid" was also used to filter out repeated instances of the same incident (which may occur, for example, when the status of an incident changes, such as when a fire is controlled). In this way, only the first report (initial emergency call) is used in our estimations.

A.2 Population density data

Population density d in the study area was provided by the Australian Bureau of Statistics. The data depicts the Usual Resident Population in 1 km^2 grid format from the 2011 Census of Population and Housing across Australia. The data offers a measure of population density (population per km^2) for Australia. The population density distribution within the study area is shown in Fig. S2, which consists of $199,805$ 1-km^2 cells, 26% of which are populated. Figure S3 shows the distribution of population amongst the cells, which illustrates a fat-tailed behavior. The fat-tailed behavior is a typical characteristic of the population density distribution across a region with large urban centers (Melbourne in this case).[1] In our study area, 93% of the cells contain a population less or equal 10 people per km^2.

A.3 Wind speed and direction data

Half-hourly measurements from the automated weather stations of the Bureau of Meteorology of Australia of wind speed and direction were used. Figure S4 shows the distribution of automated weather stations in the study area which worked during the 2009 Black Saturday wildfires. WindNinja 2.5.4[2,3] was used to generate a 3×3 km gridded wind vector field from the scattered measurements to estimate the wind speed and direction at a given location. The WindNinja model also requires temperature and digital elevation model (DEM) as inputs. Temperature measurements from the same automated weather stations, and the DEM supplied by the WindNinja program are used to run the WindNinja model.

B Calculating wind-biased outward buffers

In order to determine downwind objects, wind-biased outward buffers are calculated for previously estimated wildfire perimeters using the Huygens' principle.[4,5] As illustrated in Fig. S5, an elliptical wavelet propagates from each vertex of the previously estimated wildfire perimeters. The envelope of all elliptical wavelets determines the vertex locations of the buffer. According to,[4] the displacement (x_i^d, y_i^d) from a vertex $v_i = (x_i, y_i)$ of the previously estimated wildfire perimeter to the corresponding vertex $v_i^b = (x_i^b, y_i^b)$ of the buffer is calculated as

$$\begin{aligned} x_i^d &= c_i \sin\theta_i \\ &+ \frac{a_i^2 \cos\theta_i (x_i^s \sin\theta_i + y_i^s \cos\theta_i) - b_i^2 \sin\theta_i (x_i^s \cos\theta_i - y_i^s \sin\theta_i)}{(b_i^2 (x_i^s \cos\theta_i - y_i^s \sin\theta_i)^2 + a_i^2 (x_i^s \sin\theta_i + y_i^s \cos\theta_i)^2)^{\frac{1}{2}}} \end{aligned} \tag{1}$$

$$\begin{aligned} y_i^d &= c_i \cos\theta_i \\ &- \frac{a_i^2 \sin\theta_i (x_i^s \sin\theta_i + y_i^s \cos\theta_i) + b_i^2 \cos\theta_i (x_i^s \cos\theta_i - y_i^s \sin\theta_i)}{(b_i^2 (x_i^s \cos\theta_i - y_i^s \sin\theta_i)^2 + a_i^2 (x_i^s \sin\theta_i + y_i^s \cos\theta_i)^2)^{\frac{1}{2}}}, \end{aligned} \tag{2}$$

where x_i^s and y_i^s are the angle differentials at v_i. When the vertices of the wildfire perimeter are in clockwise order, x_i^s and y_i^s are approximated by

$$\begin{aligned} x_i^s &= x_{i-1} - x_{i+1} \\ y_i^s &= y_{i-1} - y_{i+1}. \end{aligned} \tag{3}$$

$\theta_i = \theta_i^w + \pi$ controls the orientation of the elliptical wavelet at v_i, where θ_i^w is wind direction at v_i, i.e., the direction wind blows from. a_i and b_i are half the minor axis and half the major axis of the elliptical wavelet at v_i, respectively. c_i is the distance from v_i to the center of the elliptical wavelet at v_i. a_i, b_i, and c_i are functions of wind speed U_i (km/h) at v_i.

Wind speed and direction and direction data were used to calculate θ_i, a_i, b_i, and c_i. Since the automated weather stations measure wind speed and direction every half an hour, near real-time wind speed and direction are utilized by our wildfire perimeter estimator. In,[5] a_i, b_i, and c_i are calculated as

$$\begin{aligned} a_i &= \frac{C_r(1 + C_u U_i)(1 + H_i)}{2L_i H_i} \\ b_i &= \frac{C_r(1 + C_u U_i)(1 + H_i)}{2H_i} \\ c_i &= \frac{C_r(1 + C_u U_i)(H_i - 1)}{2H_i}, \end{aligned} \tag{4}$$

where C_r and C_u are empirical coefficients; L_i and H_i are the length to breadth ratio and the head to back ratio of the elliptical wavelet at v_i. L_i and H_i are computed as[6]

$$\begin{aligned} L_i &= 0.936 \exp(0.2566 U_i) + 0.461 \exp(-0.1548 U_i) - 0.397 \\ H_i &= \frac{L_i + \sqrt{L_i^2 - 1}}{L_i - \sqrt{L_i^2 - 1}}. \end{aligned} \tag{5}$$

As the calculations at each vertex are assumed independent of the others, the resultant wind-biased buffer may contain crossovers and self-merge, which are solved in the same fashion to.[5]

C Design of experiment details

The behavior and performance of the basic algorithm (without using authoritative information) is tuned through six relatively intuitive parameters. The parameters are A_t (the minimum area of a detected fire); χ (the maximum length of any removable edge in the regular polygon representing the wildfire perimeter); τ (the size of the temporal "window" in which calls are processed); and *minPts* (the minimum number of calls), ε_s (spatial neighborhood size), and ε_t (temporal neighborhood size) used in the underlying ST-DBSCAN clustering algorithm. *minPts* can be set effectively by a simple heuristic to be the nearest integer ≥ 2 to $\ln(N)$,[7] where N is the number of calls in the temporal window. Thus the performance of the estimator is affected by the rest 5 parameters.

A full-factorial design of experiment was conducted to find the optimal parameterization and investigate the effect of the five factors on the performance of the estimator. Table S1 summarizes the notations and factorial values of the five factors in the design of experiment. The combinations of different factorial values cover an adequately large parameter space for exploring the effect of the factors on the performance of the estimator. The experiment was repeated under four settings: 1) without using authoritative information, 2) integrating wind field, 3) integrating population density, and 4) integrating both wind field and population density.

D Evaluation details

The experimental results were compared quantitatively with ground truth wildfire progression as our response variable, measuring the closeness of our results to the true wildfire perimeter. Naively, we can apply two evaluation metrics commonly-used in information retrieval: precision (positive predictive value) and recall (sensitivity).[8] Precision can be computed as the number of wildfire perimeter estimates that spatially overlap a true wildfire divided by the total number of estimates. Recall is the number of wildfires detected by our estimator divided by the total number of wildfires. However, raw precision and recall ignore the exact shape and precise location of wildfires, tending to inflate the apparent level of accuracy. Instead *area-based* precision and recall[9] is preferred in evaluating how closely the wildfire perimeter estimates track the true wildfire progression. Thus the performance of our estimator is evaluated using the area-based precision and recall.

Let $\mathbf{G}(k) = \bigcup_{i=1}^{N_f(k)} G_i(k)$ be the union of the perimeter of $N_f(k)$ wildfires burning at the kth epoch of the estimator. While our estimator generated estimated wildfire perimeters every 10 minutes, the reconstructed ground truth perimeters of multiple fires are available at less frequent and irregular intervals. To minimize this systematic effects on evaluation, the true wildfire perimeters at the kth epoch are approximated with the temporally nearest ground truth information. True positive area at the kth epoch ($A_{TP}(k)$) is defined as the area of the intersection between estimated and ground truth wildfire perimeters, i.e.,

$$A_{TP}(k) = Area\left(\mathbf{E}(k) \bigcap \mathbf{G}(k)\right). \tag{6}$$

Then area-based precision ($P_A(k)$) and recall ($R_A(k)$) at the kth epoch are defined as

$$P_A(k) = \begin{cases} \dfrac{A_{TP}(k)}{Area(\mathbf{E}(k))} & \text{if } Area(\mathbf{E}(k)) \neq 0 \\ 0 & \text{otherwise} \end{cases}$$

$$R_A(k) = \begin{cases} \dfrac{A_{TP}(k)}{Area(\mathbf{G}(k))} & \text{if } Area(\mathbf{G}(k)) \neq 0 \\ 0 & \text{otherwise.} \end{cases} \tag{7}$$

The overall area-based F1-score ($F1_A$) is used as the summary statistic of the overall performance of the estimator, computed as the harmonic mean of average precision (P_A) and recall (R_A) over all epochs,

$$F1_A = \begin{cases} \dfrac{2P_A R_A}{P_A + R_A} & \text{if } P_A + R_A \neq 0 \\ 0 & \text{otherwise.} \end{cases} \tag{8}$$

E Parameterization details

In the design of experiment under both population density and wind field weight, the optimal parameterization in Table S2 that achieves the highest overall area-based F1-score is selected. The notations and values of the factors, along with the empirical coefficients for population density and wind field weights are listed in Table S2.

F Supplementary figures and tables

```
<item>
<title>5KM SE OF RED CLIFFS, RED CLIFFS - WOOMERA AVE</title>
<link>http://www.cfa.vic.gov.au/incidents/incident_summary.htm</link>
<description>
                    &lt;b&gt;District/Region:&lt;/b&gt; MALLEE&lt;br&gt;
                    &lt;b&gt;Location:&lt;/b&gt; 5KM SE OF RED CLIFFS&lt;br&gt;
                    &lt;b&gt;Name:&lt;/b&gt; RED CLIFFS - WOOMERA AVE&lt;br&gt;
                    &lt;b&gt;Last Updated Date/Time:&lt;/b&gt; 10/09/15 01:49:00 PM&lt;br&gt;
                    &lt;b&gt;Type:&lt;/b&gt; BUSHFIRE&lt;br&gt;
                    &lt;b&gt;Status:&lt;/b&gt; GOING&lt;br&gt;
                    &lt;b&gt;Size:&lt;/b&gt; 10.00 HA.&lt;br&gt;
                    &lt;b&gt;Appliances:&lt;/b&gt; 3&lt;br&gt;
                    &lt;b&gt;Start Date/Time:&lt;/b&gt; 10/09/15 01:49:00 PM&lt;br&gt;
            </description>
<pubDate>Thu, 10 Sep 2015 03:49:00 GMT</pubDate>
<guid>1535805</guid>
<georss:collection>
<georss:point>-34.33052254116238 142.23924823633706</georss:point>
</georss:collection>
</item>
```

Supplementary Figure S1. An example of a CFA RSS feed for wildfire. The key attributes "guid", "Type", "Start Date/Time", and "georss:point" are highlighted. RSS feeds for wildfire are extracted by the attribute "Type". Status updates of existing incidents are removed by the attribute "guid". The attributes "georss:point" and "Start Date/Time" provide the spatiotemporal coordinates of the RSS feed.

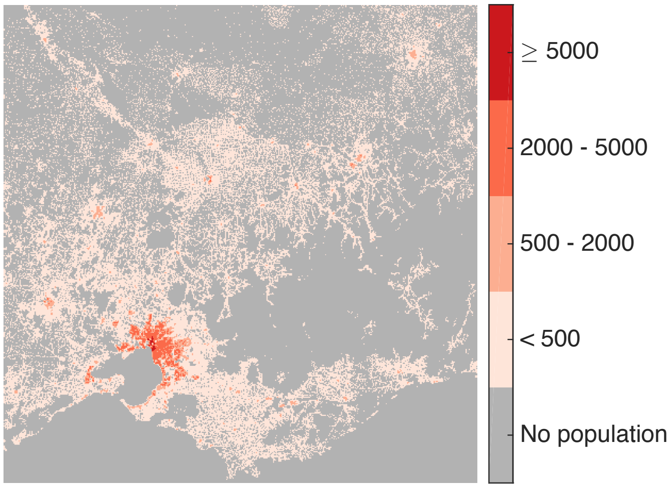

Supplementary Figure S2. Distribution of population density (people per km^2) in the study area. The map was generated using Matlab R2014b (http://au.mathworks.com).[10]

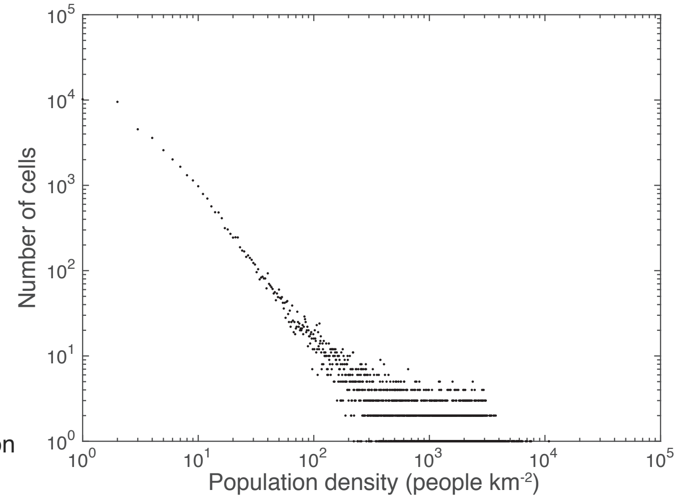

Supplementary Figure S3. Log-log plot of population distribution of cells

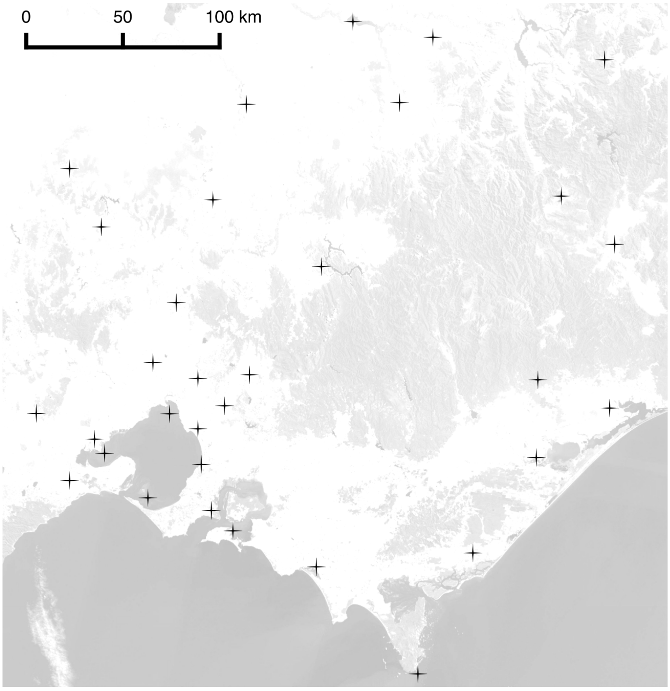

Supplementary Figure S4. Distribution of automated weather stations of the Bureau of Meteorology of Australia in the study area which worked during the 2009 Black Saturday wildfires. The map was generated using QGIS 2.8.2-Wien (`http://www.qgis.org/en/site/`)[11] and Inkscape 0.91 (`https://inkscape.org/en/`).[12]

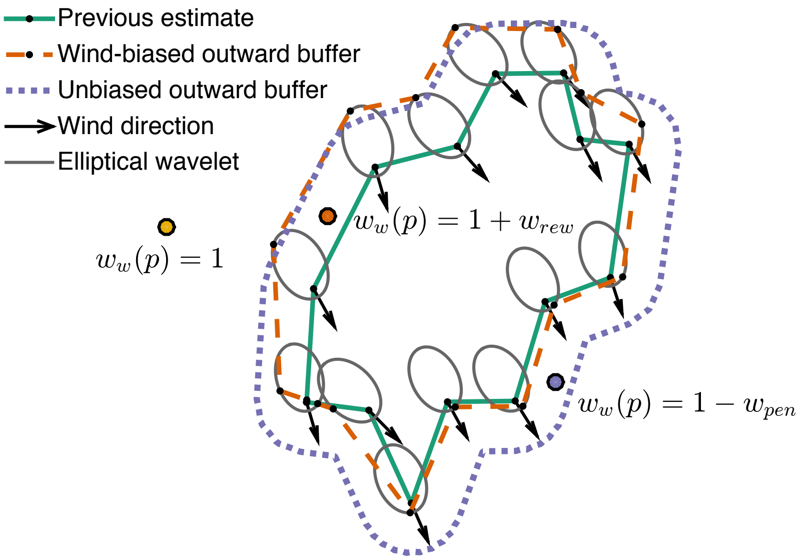

Supplementary Figure S5. Calculation of the non-uniform empirical wind field weight using Huygens' principle.

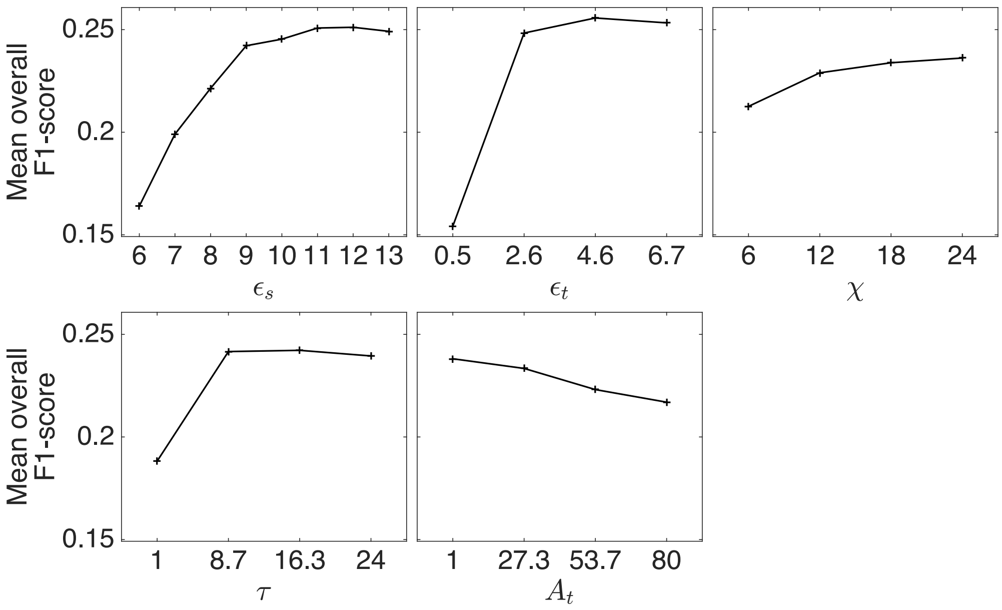

Supplementary Figure S6. Main effects of parameters upon overall area-based F1-score.

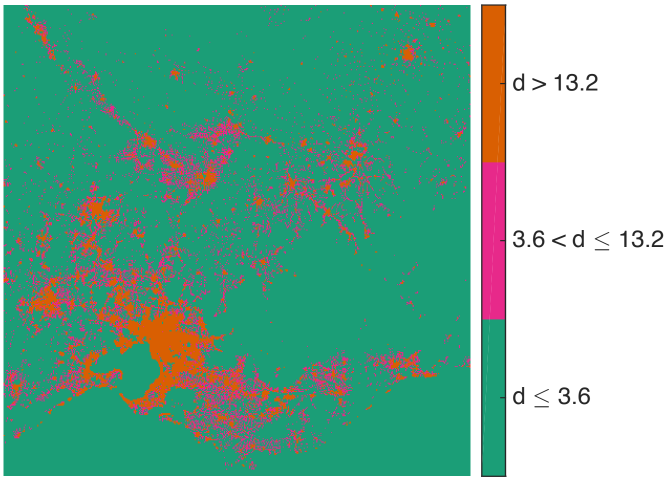

Supplementary Figure S7. Categorization of the study area into three classes of population density: $d \leq 3.6$, $3.6 < d \leq 13.2$, and $d > 13.2$. The map was generated using Matlab R2014b (`http://au.mathworks.com`).[10]

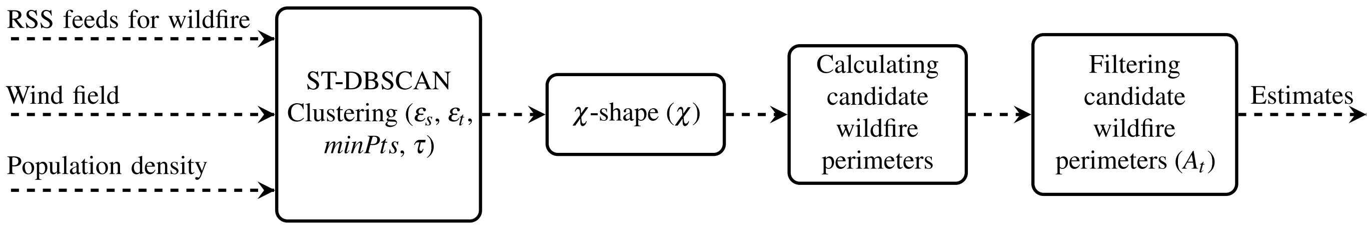

Supplementary Figure S8. Overview of the wildfire perimeter estimator.

Supplementary Table S1. The notations and experimental values of the factors in the full-factorial design of experiment.

Factor	Notation	Values
ε_s	spatial neighborhood size in ST-DBSCAN	6, 7, 8, 9, 10, 11, 12, 13 km
ε_t	temporal neighborhood size in ST-DBSCAN	0.5, 2.6, 4.6, 6.7 hours
χ	maximum length of any removable edge in the χ-shapes	6, 12, 18, 24 km
τ	the size of temporal window	1, 8.7, 16.3, 24 hours
A_t	minimum area of a detected fire	1, 27.3, 53.7, 80 km^2

Supplementary Table S2. The notations and values of all the coefficients of the wildfire periemter estimator developed in this paper.

Factor	Notation	Value	Unit
ε_s	spatial neighborhood size in ST-DBSCAN	9	km
ε_t	temporal neighborhood size in ST-DBSCAN	4.6	hours
$minPts$	minimum number of objects in the $(\varepsilon_s, \varepsilon_t)$ neighborhood of core objects	nearest integer ≥ 2 to $\ln(N)$	N/A
χ	maximum length of any removable edge in the χ-shapes	24	km
τ	the size of temporal window	16.3	hours
A_t	minimum area of a detected fire	27.3	km^2
σ_p	empirical coefficient for population density weight	331.6	km^{-2}
w_{pm}	empirical coefficient for population density weight	0.2	N/A
C_r	empirical coefficient for wind field weight	1.5	N/A
C_u	empirical coefficient for wind field weight	0.4	hour
A_w	empirical coefficient for wind field weight	120	km^2
b_w	empirical coefficient for wind field weight	13	km
w_{rew}	empirical coefficient for wind field weight	0.05	N/A
w_{pem}	empirical coefficient for wind field weight	0.9	N/A

G Legends of supplementary videos and datasets

Supplementary Video S1. Comparison video between estimated and ground truth wildfire progression. The video was generated in Matlab R2014b (`http://au.mathworks.com`)[10] by combining still images (maps) which were created using QGIS 2.8.2-Wien (`http://www.qgis.org/en/site/`)[11] and Inkscape 0.91 (`https://inkscape.org/en/`).[12]
Supplementary Dateset S1. Archived emergency call data during the Black Saturday wildfires (2009).
Supplementary Dateset S2. Archived CFA RSS feeds for wildfires during the Mickleham-Dalrymple wildfires (2014).
Supplementary Dateset S3. Population density distribution in Australia, downloaded from Australian Bureau of Statistics (`http://www.abs.gov.au/AUSSTATS/abs@.nsf/DetailsPage/1270.0.55.0072011?OpenDocument`).
Supplementary Dateset S4. Measurements of automated weather stations of Bureau of Meteorology of Australia during the Black Saturday wildfires (2009).
Supplementary Dateset S5. Measurements of automated weather stations of Bureau of Meteorology of Australia during the Mickleham-Dalrymple wildfires (2014).
Supplementary Dateset S6. Digital elevation model (DEM) in the study area, downloaded by WindNinja 2.5.4.[3]

References

1. Rutherford, A. *et al.* Limits of social mobilization. *Proceedings of the National Academy of Sciences* **110**, 6281–6286 (2013).
2. Forthofer, J., Shannon, K. & Butler, B. Simulating diurnally driven slope winds with WindNinja. In *Proceedings of 8th Symposium on Fire and Forest Meteorological Society*, 13–15 (AMS, 2009).
3. WindNinja Core Team (2016). WindNinja: A computer program for computing spatially varying wind fields for wildland fire application. Fire, Fuel, and Smoke Science Program, Logan, UT, United States. URL http://www.firelab.org/project/windninja.
4. Richards, G. D. An elliptical growth model of forest fire fronts and its numerical solution. *International Journal for Numerical Methods in Engineering* **30**, 1163–1179 (1990).
5. Finney, M. A. FARSITE: Fire area simulator: model development and evaluation. *United States Department of Agriculture, Forest Service, Research Paper* **RMRS-RP-4**, 47 (1998).
6. Alexander, M. Estimating the length-to-breadth ratio of elliptical forest fire patterns. In *Proceeding of the 8th conference on Fire and Forest Meteorology*, 287–304 (1985).
7. Birant, D. & Kut, A. ST-DBSCAN: An algorithm for clustering spatial–temporal data. *Data & Knowledge Engineering* **60**, 208–221 (2007).
8. Frakes, W. Introduction to information storage and retrieval systems. *Space* **14**, 10 (1992).
9. Nascimento, J. C. & Marques, J. S. Performance evaluation of object detection algorithms for video surveillance. *Multimedia, IEEE Transactions on* **8**, 761–774 (2006).
10. Matlab Core Team (2016). Matlab: A language of technical computing. MathWorks, Natick, Massachusetts, United States. URL http://au.mathworks.com.
11. QGIS Core Team (2016). QGIS: A free and open source geographic information system. Open Source Geospatial Foundation Project, Beaverton, Oregon, United States. URL http://www.qgis.org/en/site/.
12. Inkscape Core Team (2016). Inkscape: A free and open source professional vector graphics editor. Inkscape Project, Brooklyn, NY, United States. URL https://inkscape.org/en/.
